# Postmenopausal spontaneous uterine perforation: Case report

**DOI:** 10.4274/tjod.70370

**Published:** 2015-06-15

**Authors:** Elçin İşlek Seçen, Hilal Ağış, Canan Altunkaya, Ayşe Filiz Avşar

**Affiliations:** 1 Atatürk Training and Research Hospital, Clinic of Obstetrics and Gynecology, Ankara, Turkey; 2 Atatürk Training and Research Hospital, Clinic of Pathology, Ankara, Turkey; 3 Yıldırım Beyazıt University Faculty of Medicine, Department of Obstetrics and Gynecology, Ankara, Turkey

**Keywords:** Uterine perforation, postmenapausal, pyometra, mortality

## Abstract

Spontaneous uterine rupture and generalized peritonitis caused by pyometra occurs rarely with high morbidity and mortality. A correct and definite diagnosis can be made with laparotomy or laparoscopy. The clinical findings of perforated pyometra are similar to perforation of the gastrointestinal tract and gynecologic symptoms are less frequent, which makes preoperative diagnosis difficult. We report a case of a patient aged 82 years who underwent surgery for spontaneous uterine rupture and generalized peritonitis as a result of pyometra.

## INTRODUCTION

Pyometra is purulent fluid accumulation inside the uterine cavity as a consequence of impaired drainage^(1)^. Although pyometra is a rare disease seen in 0.1-0.2% of all gynecologic cases, it is more common in the elderly (13.6%)^(2)^. Spontaneous uterine rupture and generalized peritonitis caused by pyometra occurs rarely, approximately 50 cases have been reported in the literature to date.

We present a patient aged 82 years old, who underwent surgery for spontaneous uterine rupture and generalized peritonitis as a result of pyometra. The patient developed multiorgan failure and died on the second post operative day.

## CASE REPORT

**Figure 1:**
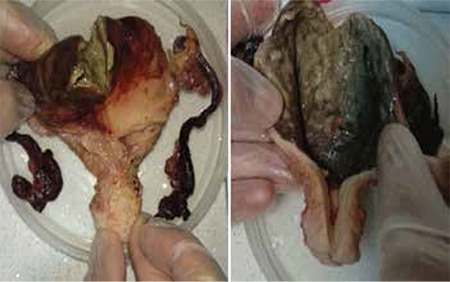
Photograph showing the perforated area and hysterectomy material

A patient aged 82 years, parity 6, who was postmenopausal for 35 years was admitted to our hospital because of abdominal pain and deterioration of general condition, which had lasted for three days. She had a history of hypertension and was a smoker of 1 pack/day. Her gynecologic history was unremarkable with no postmenopausal bleeding or discharge and she had not had an endometrial biopsy or dilatation curettage operation before. On physical examination her general condition was poor, blood pressure was 80/50 mmHg, body temperature 37.8 °C, and pulse rate was 120 beats/min. On admittance, the laboratory investigations revealed white blood cell count: 31.400/µL, hemoglobin: 10.4 g/dL, urea: 71 mgr/dL, creatinine 2.1 mgr/dL, and CRP: 195 mgr/L. The patient was in a septic condition and the abdominal computerized tomography showed generalized fluid and free air; abdominal purulent fluid was obtained via paracentesis by the surgery department. An emergency laparotomy was performed with the diagnosis of gastrointestinal perforation. There was approximately 1000 cc purulent fluid in the abdominal cavity, further exploration revealed a 2.5-cm-long perforation at the anterior of the uterus (Figure 1) from which purulent drainage continued. Other abdominal organs were normal and emergency gynecology consultation was acquired. The uterus was necrotic and showed no signs of malignancy. Total abdominal hysterectomy with bilateral salpingo-oopherectomy was performed and histopathologic examination revealed ischemic necrosis and chronic cervicitis.

The patient was treated in the intensive care unit, ceftriaxone 0.5 g bid and metranidazole 0.5 g tid was administered. On the first postoperative day, liver function tests showed an abrupt increase (alanine transaminase: 392 U/L, aspartate transaminase: 1517 U/L), like kidney functions (urea: 72 mgr/dL creatinine: 2.4 mgr/dL).

The patient’s general condition continued to deteriorate and she died on the second post operative day of multiple organ dysfunction syndrome.

## DISCUSSION

Pyometra, although rare in the general population, is found more frequently in postmenopausal women as result of conditions like occlusion of the cervical channel by malignant or benign tumors, surgery, radiotherapy, and senile cervicitis^(3)^. The classical triad of symptoms includes postmenopausal vaginal bleeding, purulent vaginal discharge, and suprapubic pain^(4)^. However, more than 50% of all cases are asymptomatic^(5)^. Our patient had abdominal pain for three days but had no postmenopausal bleeding or discharge.

Spontaneous uterine rupture occurs rarely and shows two incidental peaks. It occurs due to pregnancy or intra uterine devices in the reproductive period and as a result of pyometra postmenopause^(6)^. The frequent symptoms of uterine perforation due to pyometra include abdominal pain (97.6%), fever (54.8%), and vomiting (31%)^(7)^. Although atrophic endometrium is a common cause, perforation is usually seen in the presence of serious causes such as cervical or endometrial carcinoma or a forgotten intrauterine device. Malignant disease is present in 35% of cases^(1)^. Our patient had no evidence of malignancy during surgery and at pathology, she had no intrauterine device, and had not undergone endometrial biopsy or dilatation curettage operations before. Therefor, the most probable cause of pyometra was postmenopausal changes and stenosis of the cervix. Uterine perforation is usually seen at the fundus (77%), but may occur anteriorly (4%)^(7)^.

Preoperative diagnosis of uterine perforation due to pyometra is difficult. Imaging modalities show pneumoperitoneum and intrabdominal fluid, which frequently leads to the misdiagnosis of gastrointestinal perforation^(5)^. Sagittal and coronal reformats in multi-detector computerized tomography are very helpful in depicting the site and size of uterine breach by demonstrating the resultant intra-abdominal collections thus playing an important role in the diagnosis of ruptured pyometra. Sonography has limited use in the diagnosis of ruptured pyometra because of its inability to demonstrate the uterine breach and the limited sonographic window available due to perforation^(8)^. However, correct preoperative diagnosis is made in 30% of patients^(7)^. In our patient, abdominal computerized tomography showed generalized fluid and free air; abdominal purulent fluid was obtained via paracentesis by the surgery department. An emergency laparotomy was performed with a diagnosis of gastrointestinal perforation owing to the patient’s age and no gynecologic symptoms.

The mortality rate of uterine perforation caused by pyometra is 15%. In the majority of patients the cause of death is multiple organ dysfunction syndrome due to sepsis^(7)^. Despite the interventions, our patient died on the second postoperative day of multiple organ failure syndrome and sepsis. The patient sought medical advice on the third day of her complaints and was already in septic shock and was a smoker, both of which probably contributed to her poor prognosis.

Uterine rupture caused by pyometra is serious and occurs rarely with high morbidity and mortality. The patients present with diffuse abdominal peritonitis and gynecologic symptoms are less frequent, which makes preoperative diagnosis difficult. Uterine rupture should be borne in mind in postmenopausal patients who present with acute abdomen and pneumoperitoneum.
